# New insights on partial trisomy 3q syndrome: *de novo* 3q27.1-q29 duplication in a newborn with pre and postnatal overgrowth and assisted reproductive conception

**DOI:** 10.1186/s13052-023-01421-y

**Published:** 2023-02-09

**Authors:** Gregorio Serra, Vincenzo Antona, Marcello Cimador, Giorgia Collodoro, Marco Guida, Ettore Piro, Ingrid Anne Mandy Schierz, Vincenzo Verde, Mario Giuffrè, Giovanni Corsello

**Affiliations:** Department of Health Promotion, Mother and Child Care, Internal Medicine and Medical Specialties “G. D’Alessandro”, University of Palermo, Palermo, Italy

**Keywords:** Chromosome 3, Contiguous gene syndrome, Prenatal diagnosis, ART, a-CGH, Case report

## Abstract

**Background:**

Duplications of the long arm of chromosome 3 are rare, and associated to a well-defined contiguous gene syndrome known as partial trisomy 3q syndrome. It has been first described in 1966 by Falek et al., and since then around 100 patients have been reported. Clinical manifestations include characteristic facial dysmorphic features, microcephaly, hirsutism, congenital heart disease, genitourinary anomalies, hand and feet abnormalities, growth disturbances and intellectual disability. Most of cases are due to unbalanced translocations, inherited from a parent carrying a balanced aberration (reciprocal translocation or inversion), and rarely the genomic anomaly arises de novo. Very few studies report on the prenatal identification of such rearrangements.

**Case presentation:**

Hereby, we report on a newborn with a rare pure duplication of the long arm of chromosome 3. Noninvasive prenatal test (cell free fetal DNA analysis on maternal blood), performed for advanced parental age and use of assisted reproductive technique, evidenced a partial 3q trisomy. Then, invasive cytogenetic (standard and molecular) investigations, carried out through amniocentesis, confirmed and defined a 3q27.1-q29 duplication spanning 10.9 Mb, and including about 80 genes. Our patient showed clinical findings (typical facial dysmorphic features, esotropia, short neck, atrial septal defect, hepatomegaly, mild motor delay) compatible with partial trisomy 3q syndrome diagnosis, in addition to pre- and postnatal overgrowth.

**Conclusions:**

Advanced parental age increases the probability of chromosomal and/or genomic anomalies, while ART that of epigenomic defects. Both conditions, thus, deserve more careful prenatal monitoring and screening/diagnostic investigations. Among the latter, cell free fetal DNA testing can detect large segmental aneuploidies, along with chromosomal abnormalities. It identified in our patient a wide 3q rearrangement, then confirmed and defined through invasive molecular cytogenetic analysis. Neonatologists and pediatricians must be aware of the potential risks associated to duplication syndromes. Therefore, they should offer to affected subjects an adequate management and early and careful follow-up. These may be able to guarantee to patients satisfactory growth and development profiles, prevent and/or limit neurodevelopmental disorders, and timely recognition of complications.

## Background

Duplications of the long arm of chromosome 3 are rare, and usually diagnosed after birth [1]. They are associated to well-defined conditions known as partial trisomy 3q syndrome [2]. Its clinical manifestations include typical dysmorphic facial features (synophrys, broad nasal bridge, anteverted nares, micrognathia, low-set dysplastic ears), microcephaly, hirsutism, congenital heart disease, genitourinary disorders, growth disturbances and cognitive deficits [2]. In around 70% of cases, the genomic anomaly is the result of a balanced translocation of a parent, who is carrier of a concomitant deletion of another chromosome, while in the others it arises de novo [3]. In 1966, Falek et al. [4] described the first subject affected with partial duplication 3q, and since then around 100 patients have been reported [5]. The critical region associated with the syndrome phenotype was later mapped to 3q26.3q27.7 [6, 7]. Cases with pure and more distal duplications have been rarely reported, and even more uncommon are those identified through prenatal diagnostic tests [8]. Hereby, we report on a newborn with prenatal finding of a de novo 3q27.1q29 duplication of 10.9 Mb, identified and defined with array comparative gemomic hybridization (a-CGH) analysis, performed through amniocentesis due to advanced parental age and use of assisted reproductive technique (ART, namely egg donation and intracytoplasmatic sperm injection, ICSI), and clinical manifestations compatible with partial trisomy 3q syndrome diagnosis, in addition to pre- and postnatal overgrowth.

## Case presentation

**Fig. 1:**
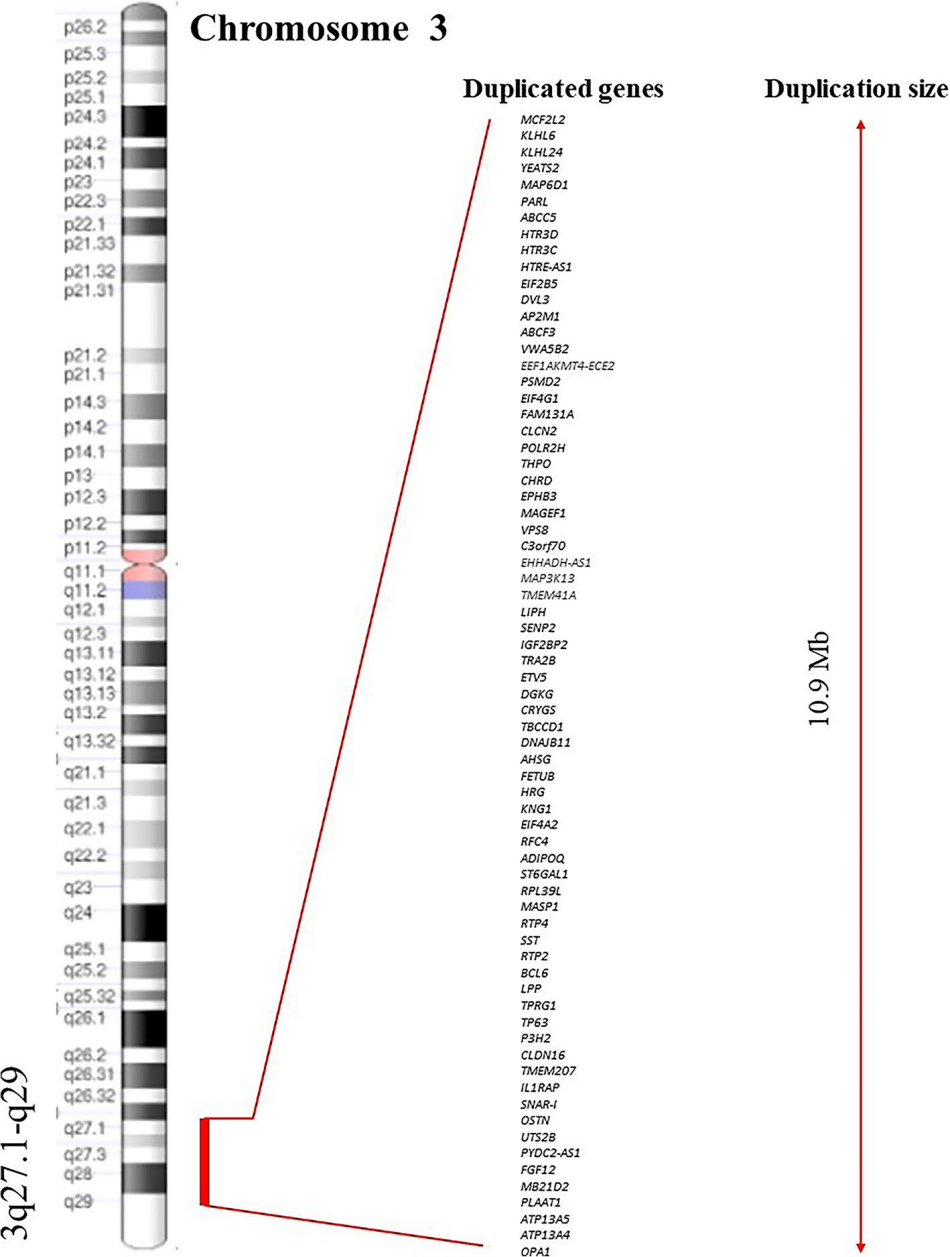
Overview of chromosome 3, showing present patient’s q27.1-q29 duplication and involved genes, spanning 10.9 Mb of genomic DNA from position 182,989,731 to 193,854,071, according with DECIPHER Genome Browser (GRCh37/hg19 assembly) [12]

**Fig. 2:**
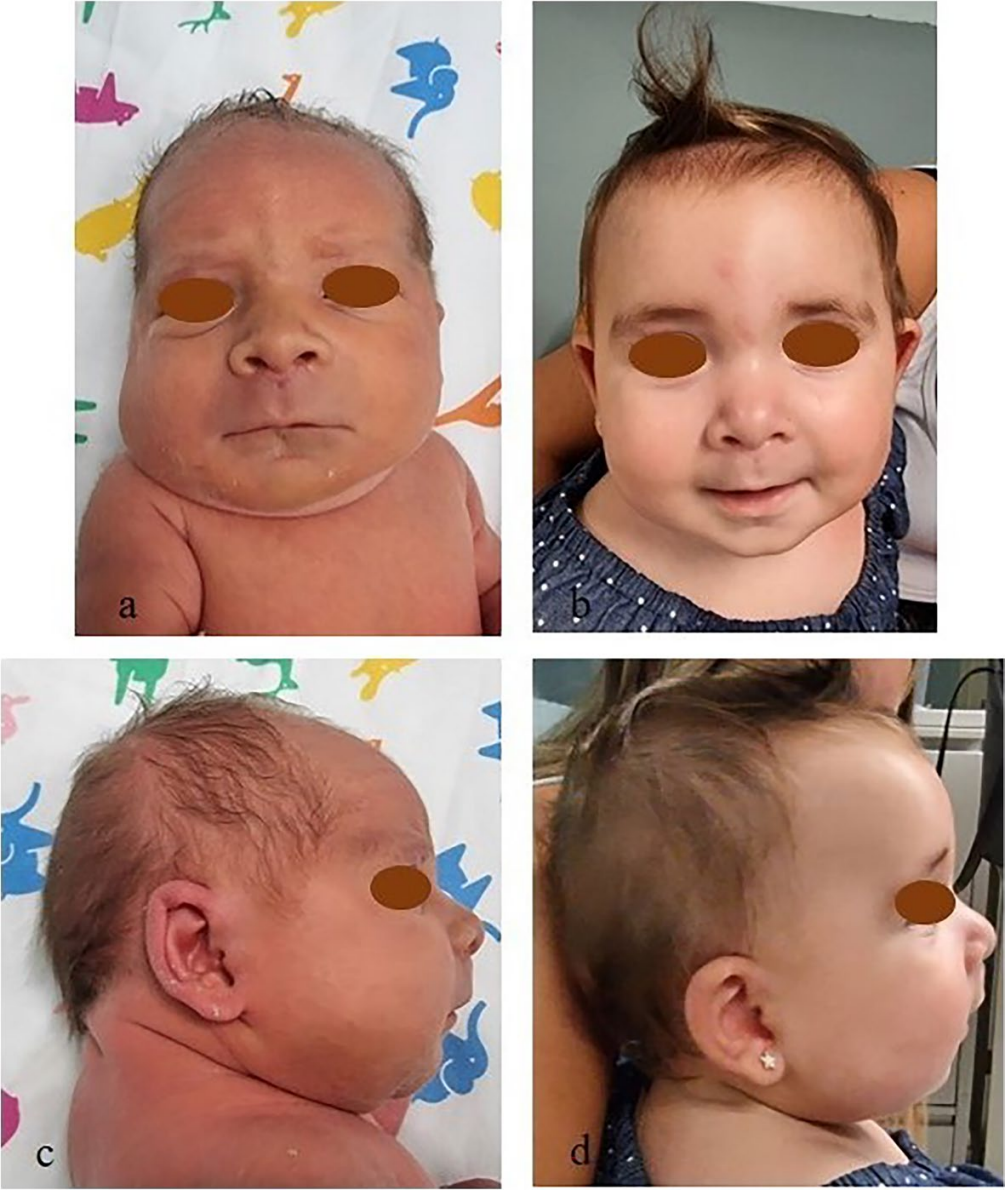
**a/b/c/d a** and** b** Patient’s front view at birth and age 8 months: high frontal hairline, bushy eyebrows, down slanting palpebral fissures, broad nasal bridge, bulbous tip, anteverted nares, long philtrum, large maxilla, carp shaped mouth with thin lips and downturned corners. **c** and** d** Lateral view at birth and age 8 months: short and wide neck, low-set, dysplastic and anteriorly rotated ears with prominent helix, and microretrognathia

**Fig. 3:**
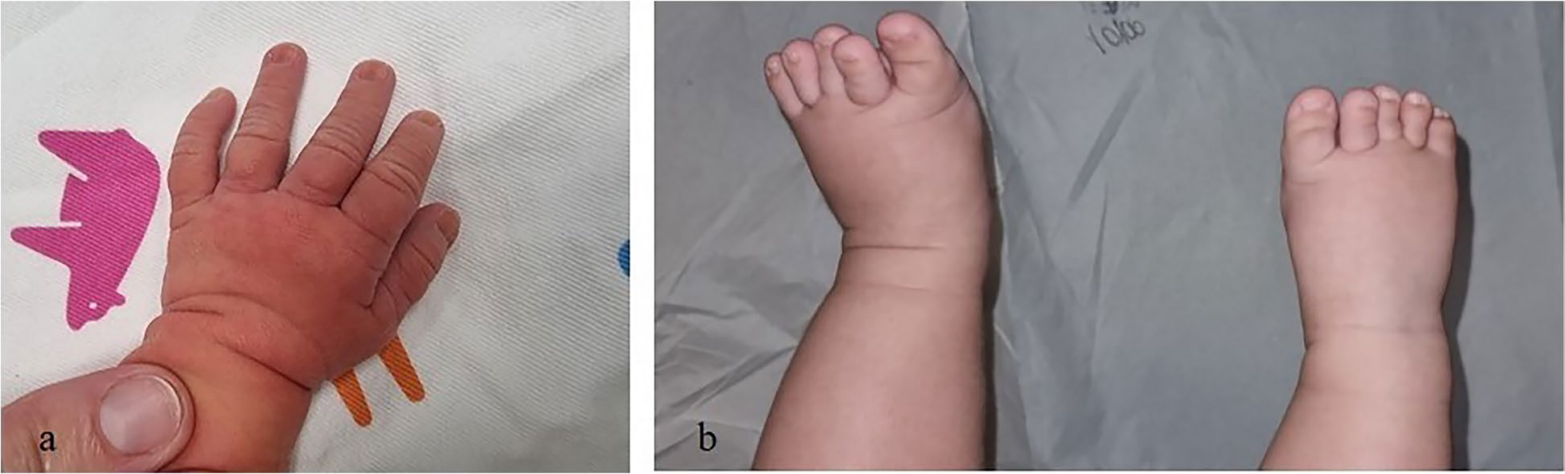
**a/b** **a** Brachydactyly of fingers, with proximally placed thumb and clinodactyly of the fifth one. **b** Feet brachydactyly with bilateral *hallux varus* and crowded toes (overlapping of the second and fourth toes on the third and fifth ones)

A female newborn was the first child of healthy and nonconsanguineous parents (46 year-old mother and 42 year-old father). She was delivered preterm at 35 + ^2^ weeks of gestation (WG) from the third pregnancy, by cesarean section due to metrorrhagia complicating placenta previa. Family history was negative for genetic diseases and/or malformation syndromes. Two previous pregnancies ended with miscarriages occurred during the first trimester. Current gestation was obtained by medically ART (in-vitro fertilization treatment by egg donation and intracytoplasmatic sperm injection, ICSI). Obstetric history revealed increased nuchal translucency (4.7 mm) on the first trimester screening examination. Soon after, non-invasive prenatal testing (NIPT), using cell free fetal DNA analysis in maternal blood, evidenced a duplication of the long arm of chromosome 3. Therefore, invasive diagnostic investigations through amniocentesis, including standard and molecular cytogenetic examinations, were suggested by obstetricians [9] and performed by the mother, along with subsequent genetic counselling. Karyotype test identified a normal female set, and a partial duplication involving the long arm of chromosome 3 (46,XX,dup(3) (q27.1q29), which was then confirmed by a-CGH analysis. The latter further defined the anomaly, spanning 10.9 Mb from position 182.989.731 to 193.854.071 (Oligo array platform 8 × 60 K, mean resolution 200–250 kb, genome assembly GRCh37.p13). The genomic abnormality comprises about 80 genes, some of which are disease causing and included in the minimal critical region responsible for 3q duplication syndrome (i.e. *DVL3*, *EPHB3*) (Fig. 1). a-CGH and fluorescent in situ hybridization (FISH) were, then, also performed in the father (the normal standard and molecular cytogenetic profiles of the biological mother were ascertained before egg donation) and evidenced no segmental aneuploidies and/or structural chromosomal rearrangements at the 3q27.1q29 region. Genetic consultation, then, confirmed that the observed genetic profile was compatible with the indication (NIPT detection of chromosome 3 abnormality) for which the fetal diagnostic test had been requested. Pregnancy follow-up through second level prenatal ultrasound (US) investigations was also suggested. Finally, the genetic tests performed in the father and the mother and resulted normal, allowed to provide a reproductive counselling to the family, and to establish the recurrence risk as low. Subsequent US investigations performed during the second trimester (21 WG), revealed increased fetal growth (main anthropometric measures around the 90^th^ centile), in addition to lumbosacral lordosis and bilateral first degree hydronephrosis. Apgar scores were 7 and 7, at 1 and 5 min. Postnatally, our patient needed primary resuscitation, which was briefly conducted through non-invasive positive pressure ventilation. Due to dysmorphic features, along with prenatal findings of genetic and renal abnormalities, she was transferred on the first day of life to our Neonatal Intensive Care Unit. At admission, anthropometric measures were as follows: weight 2900 g (92^nd^ centile, + 1.43 standard deviations, SD), length 47 cm (74^th^ centile, + 0.64 SD), occipitofrontal circumference (OFC) 33.5 cm (89^th^ centile, + 1.23 SD). Physical examination disclosed high frontal hairline, bushy eyebrows, long eyelashes, down slanting palpebral fissures, telecanthus, epicanthus, broad nasal bridge, bulbous tip, anteverted nares, long philtrum, large maxilla, carp shaped mouth with thin lips, downturned corners and tendency to keep it open (Fig. 2a). Short and wide neck, low-set, dysplastic and anteriorly rotated ears with prominent helix, and microretrognathia completed the craniofacial profile (Fig. 2c). Abdominal evaluation disclosed palpable liver, 3 cm under the costal arch. Bilateral brachydactyly of fingers, with proximally placed thumbs and clinodactyly of the fifth ones, as well as feet brachydactyly with bilateral *hallux varus* and crowded toes (overlapping of the second and fourth toes on the third and fifth ones, respectively) were also observed (Fig. 3a/b). Neurological findings were a mild axial central type hypotonia, as well as decreased osteotendinous and archaic reflexes. Due to the mild perinatal hypoxic injury and the following respiratory distress, she performed non-invasive ventilatory support during the first 72 h of life, and then oxygen supplementation for further two days. Owing to lack of the sucking reflex, nasogastric tube feeding along with parenteral nutrition were initially required and continued until the third day of life. Then, gradual improvement of enteral nutrition allowed adequate and complete bottle feeding, with standard infant formula [10], from day 14 of life. Newborn metabolic screening along with blood and urine biochemical tests showed normal results. Head US detected asymmetric ventricular system due to mild enlargement of the left lateral ventricle. Abdomen US revealed a bilateral II degree hydronephrosis (according with radiology grading system), with renal pelvis dilation, in the anteroposterior diameter, of 20 and 18 mm of the left and right kidney respectively, as well as hepatomegaly with homogenous echo structure. Echocardiogram documented an *ostium secundum* type atrial septal defect (ASD), in addition to patent *foramen ovale*. Ophthalmological evaluation documented no abnormalities. The following clinical course was regular, and only marked by global overgrowth. She was then discharged, and included in a multidisciplinary (auxological, neurodevelopmental, cardiological, ophthalmological, audiological, urological/surgical) follow-up. Serial auditory brainstem response (ABR) evaluations, at 2 and 4 months of age, ruled out hypoacusis. In the following months, heart US evaluations excluded the persistence of *foramen ovale*, while renal US documented a progressive decrease of hydronephrosis, lasting only in the left kidney (anteroposterior diameter of the renal pelvis 15 mm). Head US no longer showed abnormalities, while ophthalmological evaluation conversely disclosed bilateral esotropia. No renal and/or urinary tract infections have been registered to date. The patient currently is 8 months and 6 days old (7 months and 3 days of corrected age), and shows, according to World Health Organization growth standards for neonatal and infant close monitoring [11], global overgrowth: weight 10.1 kg (99^th^ centile, + 2.2 SD), length 73 cm (99^th^ centile, + 2.3 SD), and OFC 46.2 cm (99^th^ centile, + 2.5 SD) (Fig. 2b/d). Neurodevelopmental assessment is at present normal in relational, communication, fine and gross motor areas with normal righting reactions, apart a mild delay involving upper limb lifting in prone position. She presently shows no further abnormalities.

## Discussion and conclusions

Partial duplication 3q syndrome is a rare but well-defined clinical entity [1, 2]. Its phenotype includes abnormalities of the central nervous system, typical facial dysmorphic features, congenital heart disease, urogenital tract, hands and feet abnormalities, growth disturbances and intellectual disability [1]. It must be distinguished from other genetic diseases, mostly from Cornelia de Lange syndrome [5]. It is usually diagnosed after birth [1]. The present case is among the very few reported ones identified in utero [1, 8]. The critical genomic region responsible for dup(3q) syndrome phenotype has been narrowed down to 3q26.3-q27.7 regions [1, 3, 5, 13]. However, ours is among the rarely described cases with more distal duplication including chromosomal bands telomeric than q27 [2]. Indeed, the bands from 3q26.3 to 3q27 are spared in our patient, who however shows most of the clinical manifestations of the syndrome, suggesting that a role for the related critical genes might be narrowed down between 3q27.1 and 3q27.3. Actually, the comparison of our patient with those, among the few reported in literature, with even rarer “pure” 3q duplications and available precise genomic and clinical characterization reveals typical common findings, which are summarized in Table 1.

**Table 1:** Comparison of present patient phenotype with that of previously described “pure” 3q24q29 duplications (modified by Grossmann et al., 2009 [2])

Clinical manifestations	dup 3q24-q26.31, *n* = 1, Meins et al. 2005 [14]	Dup 3q25-29, *n* = 2, Wilson et al., 1978 [15]	Dup 3q29, *n* = 8, Ballif et al. 2008, Lisi et al. 2008 [16, 17]	Dup 3q26.3, *n* = 2, Azar et al. 1999 [18], Faas et al. 2002 [13]	Dup3q25-qter, *n* = 11, ECARUCA [19]	Dup3q27-qter, *n* = 6, ECARUCA [19]	Dup3q27.1-q29(Present patient)
Growth retardation							
Prenatal			2/5		1/1	2/2	
Postnatal			2/5	1/ 2			
Overgrowth							+
Microcephaly	1/1	2/2	5/8		4/11		
Macrocephaly			1/8				+
Dysmorphic facial features							
Low frontal hairline		2/2	1/8	1/ 2	2/11		+
High frontal hairline				2/2			+
Bushy eyebrows				5/11			+
Hypertelorism					4/11	3/6	+
Downslanting palpebral fissures	1/1		3/8	1/2	5/11	1/6	+
Epicanthus	1/1				4/11	3/6	+
Wide nasal bridge		1/2	4/8	2/2	6/11	2/6	+
Bulbous nasal tip	1/1	2/2		2/2	9/11		+
Prominent philtrum	1/1			1/2	5/11		+
Large or downturned corners of the mouth	1/1	1/2	3/8	1/2	6/11		+
Low-set dysplastic ears	1/1			1/2	4/11	4/6	+
Hirsutism		2/2		1/2	7/11	1/6	
Hands and feet abnormalities							
Brachydactyly							+
V finger clinodactyly		2/2			5/11	1/6	+
Heart defects	1/1		1/8	1/2	1/11		+
Urogenital anomalies					2/11	1/6	+
Developmental delay	1/1	2/2	6/8	2/2	5/11	4/6	+ (mild motor delay)

+  = present

In the attempt to establish if a genotype–phenotype correlation exists, comparing our patient with the previously reported ones carrying more proximal duplications, brain abnormalities leading to cognitive disability and epilepsy have been more commonly observed in these subjects [1, 3]. The duplicated chromosomal fragment 3q26.3-q27.7 contains different genes (e.g., *NLGN1*, *BCHE*, *TNIK*, *SOX2* and *Map6D1 *) highly expressed during the embryonic development of the brain [1, 5], many of which are spared in the rearrangement of our proband. This may explain the more expressed neurological alterations of these patients [1, 3], compared with the minimal neurodevelopmental involvement of the *proposita*, mildly affecting only the psychomotor domain. Moreover, some patients with dup(3q) and caudal defects (one patient affected by Currarino syndrome) have been described [5], suggesting that these anomalies may be included in the phenotype of the syndrome. However, no caudal abnormalities have been observed in the present patient. Also, prenatal growth retardation is more frequently present in cases with more proximal breakpoints in 3q [2]. Conversely, normal postnatal growth, or even increased as in our case and in those reported by Grossmann et al. [2] and Lisi et al. [17], seems to be associated with a more distal breakpoint. Therefore, a chromosomal region associated to growth delay is likely located proximal to band 3q27.1, while genes responsible for growth excess, macrocephaly and obesity may harbor within the rearranged chromosomal region of our patient, according to literature data [2, 17]. Finally, the copy number variation (CNV) here reported involves the 3q29 region associated with moderate cognitive deficits, and other abnormalities (defining the 3q29 microduplication syndrome, MIM #611,936) [5, 16, 17, 20], which could also contribute to the phenotype of our proband.

An overall careful comparison of the clinical features described in other 3q duplications can help clarify the influence of specific genomic regions on the phenotype. Then, the phenotypic variability between the patients reported to date can be explained by variations of the number (and type) of active genes present in chromosomal fragments of different sizes, according to contiguous gene syndromes [3, 21–27]. Indeed, the genes included in the present duplicated chromosome are around 80, and some of them are associated with well-known phenotypes of the 3q duplication syndrome [2]. Among them, there are some disease causing ones, including *DVL3* (MIM #601,368), *EPHB3* (MIM #601,839), *IGF2BP2* (MIM #608,289), *AHSG* (MIM #138,680), *ADIPOQ* (MIM #605,441), *LPP* (MIM #600,700), *TP63* (MIM #603,273), *P3H2* (MIM #610,341), *FGF12* (MIM #601,513), and *OPA1* (MIM #605,290) (Fig. 1) [28]. Two (*DVL3* and *EPHB3*) are those comprised within the partial trisomy 3q syndrome critical region [5], while the others are involved in many processes, including cellular growth, brain, heart, skeletal, skin tissues and urorectal development [5], and their mutations are mainly associated with neurological, cardiac, and ocular diseases, as well as to some types of cancer [29].

Advanced parental age increases the probability of chromosomal and/or genomic anomalies, while ART that of epigenomic defects [30, 31]. Both conditions, thus, deserve more careful prenatal monitoring and screening/diagnostic investigations. Among the latter, cell free fetal DNA analysis may have a key role. Actually, it allowed in our patient the identification before birth of a large rearrangement of chromosome 3q, then confirmed and defined (precise determination of size and gene content) through molecular karyotyping technique. The present case shows how minor phenotypic effects may be due to large chromosomal rearrangements, especially if these are duplications compared to deletions. The genetic characterization of such conditions, as occurs in other chromosomal and/or genomic diseases, may address clinicians towards more individualized follow-up [32–41], focusing on definite issues (e.g., auxological and nutritional aspects, ophthalmological assessment, oncologic surveillance), in relation to the involvement of genes potentially linked to the development of specific diseases (i.e., obesity, strabismus, cancers) [5, 14, 42, 43].

The present study may be helpful to a better characterization of both clinical and genomic features of 3q duplication syndrome. It underlines how neonatologists and pediatricians must be aware of the potential risks associated to such conditions. Therefore, they should offer to affected subjects an adequate management and early and careful follow-up. These may be able to guarantee to patients satisfactory growth and development profiles, prevent and/or limit neurodevelopmental disorders, and timely recognition of complications [44–50].

## Data Availability

The datasets used and analyzed during the current study are available from the corresponding author on reasonable request.

## Abbreviations

a-CGH: Array-Comparative Genomic Hybridization
ART: Assisted Reproductive Techniques
ASD: Atrial Septal Defect
CNV: Copy Number Variation
FISH: Fluorescent In Situ Hybridization
ICSI: Intracytoplasmic Sperm Injection
NIPT: Non-Invasive Prenatal Testing
OFC: Occipitofrontal Circumference
TEOAEs: Transient-Evoked Otoacoustic Emissions
US: Ultrasound
WG: Weeks of Gestation
